# Does the Use of Social Media Tools in Classrooms Increase Student Commitment to Corporate Social Responsibility?

**DOI:** 10.3389/fpsyg.2020.589250

**Published:** 2020-12-23

**Authors:** Sara Rodríguez-Gómez, Raquel Garde-Sánchez, María Lourdes Arco-Castro, María Victoria López-Pérez

**Affiliations:** Departamento de Economía Financiera y Contabilidad, Facultad de Ciencias Económicas y Empresariales, Universidad de Granada, Granada, Spain

**Keywords:** Corporative Social Responsibility, business ethics, higher education, digital technologies, social media

## Abstract

There is an increasing demand for ethical and Corporate Social Responsibility (CSR) practices by companies. This competence has to be introduced in students’ training in business degree programs, and a check must then be done to determine if the students have come to appreciate the importance of CSR commitments. Using the framework of Stakeholders Theory, this work aims to examine students’ perceptions of ethical and CSR practices and commitment to different stakeholders, as well as the factors that lead students to act in a socially responsible way. Furthermore, we hope to identify how the perception of CSR can be improved when Web 2.0 and social media tools that have proven effective in transmitting emotions and values are used in classrooms to teach these ideas. To this end, a survey was carried out in the year 2019 with 1,030 first-year students; it was administered at the beginning of the semester and also at the end of the semester after the training activities had been carried out. The main finding of the research is that students start with the belief that ethics and CSR are developed for reasons of image and legitimacy; however, after receiving training on these topics through tools that take into account emotions and values, they start to value the importance of the company as an agent of social change. The main practical and managerial implication is that methods based on Web 2.0 and social media tools are useful to teach ethics and CSR; the theoretical contribution is that students take into account the welfare of others. This finding contributes to Stakeholder Theory in a higher education context.

## Social Media in CSR Training in Undergraduate Studies

The financial scandals of the early 2000s have led to greater demand for ethical behavior in the business world (Brunton and Eweje, 2010). The demand for Corporate Social Responsibility (CSR) necessitates the training of students to solve CSR-related problems to help them to identify how and why to address those problems (Hosmer, 2006; Brunton and Eweje, 2010).

Universities must provide the necessary skills and knowledge to determine the social, ethical and environmental effects of business activities (Brampton and Maclagan, 2005). Thus, in curricular design, universities should integrate aspects of ethics and social responsibility (Nelson et al., 2012; Stonkutë et al., 2018). It is necessary to know students’ beliefs about the ethical commitments of companies when they arrive at university in order to determine the most appropriate teaching tools to instruct them in social responsibility. Specifically, we propose the use of social media in the early stages of ethical training, which has been understudied and about which there is a considerable knowledge gap.

In this sense, the aim of this article is, on the one hand, to examine students’ perceptions of companies’ ethical and CSR practices, commitments and reasons, and, on the other hand, to see how this perception can be improved when social media tools are used in classrooms to transmit and discuss concepts using case studies and readings related to ethics and CSR. We look at CSR to examine how technology might affect student performance, specifically the process of learning ethical competence. The knowledge that university students acquire affects how they identify the main ethical and CSR commitments that a company must assume (Holland and Albrecht, 2013). Instruction in this area leads students to recognize the firms’ commitment to different stakeholders and to know the reasons that have led the companies to act according to ethics and CSR. The main contributions of this work lie in providing the literature with data on the most efficient methodologies and tools for effectively teaching concepts related to ethics and CSR for students starting their undergraduate studies.

The role of the instructor in the teaching–learning process is essential for the planification, transmission, and acquisition of students’ knowledge and skills (Serrano and Pons, 2011). To perform this role correctly, the instructor must be able to apply methodological strategies (e.g., learning and assessment) appropriate to the students’ needs and to use Information and Communication Technologies (ICT) to help improve teaching–learning processes (Triadó et al., 2014). The instructor analyses the participants’ answers to cases to see the factors on which the students focus their attention, as these are the critical elements that must be addressed in order to guide the presentation of the cases to encourage a more socially responsible vision for students in which they make ethical decisions. The focus is on the students, the group, the networks in which they interact, their participation and the way they are invited to participate in those networks (Berthon et al., 2012); this is necessary for any contribution to have value in bolstering our understanding of the complex problems that arise from CSR.

In CSR, participants are not recipients of training but producers of it. It is important to point out that the participants are the driving force in this media world; hence, it is critical that the instructor perfectly know the tools and their use to achieve the purpose of their ethical training, which facilitates appropriate decision-making in CSR actions. Again, participants are not recipients of training but producers of it.

Based on the knowledge they have been taught, university students identify the main ethical and CSR commitments that a company must assume (Holland and Albrecht, 2013). Stakeholders Theory is the most widely used theory in the business world (Freeman and Reed, 1983). This theory takes into account the different expectations and demands of society and stakeholders (Carroll, 1979; Arco-Castro et al., 2020) and considers the role of concern for others. This is the approach taken in teaching. The training that is provided should have an impact on students, and their attitudes and beliefs should change as a result of the training (Cohen et al., 2001). Training in business ethics can bring about a change in personal values and attitudes (Balotsky and Steingard, 2006).

Web 2.0 tools and social media have a place in the field of education. Their use enables students to acquire professional competencies (Lei, 2010). University education should promote the use of social networks insofar as doing such enables and facilitates communication, cooperation, interaction, and the exchange of knowledge (Aral et al., 2013; Sigala and Chalkiti, 2015). The skills required in business go beyond formal classroom instruction, which indicates the need for the development of new teaching methodologies, where, with the introduction of blended learning techniques (López-Pérez et al., 2011), Web 2.0 tools have a place (Rae, 2010). This mainly takes the form of social networks, although wikis or concept maps are also used. Education must incorporate methodologies that introduce flexible technological tools that enhance and enable creativity, dialogue, curiosity, and emotion, as well as their application to personal and real-world problems and opportunities (Rae, 2010).

These types of technological tools may be relevant in the teaching of ethics and CSR, since learning ethics includes emotions, values (Rodriguez-Gomez et al., 2020), and social interaction, and teaching methods based on Web 2.0 can be helpful given that social media tools have proven effective in those areas (Shrivastava, 2010; García-Morales et al., 2020). It is important to point out that creative participants are the driving force in this media world, hence, the importance of the teacher knowing the tool and its use, so that the tool can serve a purpose in ethical training that facilitates appropriate decision-making in CSR actions.

We use ICT to refer to technology that has been developed specifically to reinforce academic content as a support for face-to-face instruction. We examine how such technology might affect student performance, specifically how it affects the learning process of ethical competence, by looking at CSR (López-Pérez et al., 2011; Montiel et al., 2018; García-Morales et al., 2020). In this sense, we propose the following research hypotheses:

H1: Social media improves students’ perception of business ethics and CSR.
H2: Social media improves students’ opinions about business CSR commitments.
H3: Social media improves students’ understanding of why companies act in a socially responsible way.

## Method

### Sample Selection

Our work aims to analyze university students’ perceptions of CSR and ethics issues in their first-year of university studies and to consider the evolution that this perception undergoes when this content is taught using social media tools. A survey was carried out on first-year students matriculated in an introduction-level financial accounting course at a university in Andalusia in 2019. The class had a total enrollment of 1,030 students.

For the selection of the sample, we only considered students who attended at least 80% of the classes. We obtained a total of 510 valid questionnaires at the beginning of the semester (49.51% of those enrolled), which made up the final sample (sampling error 3.15%, confidence level 95.5%, *Z* = 1.96, *p* = *q* = 0.5). In addition, 374 valid questionnaires were obtained at the end of the semester (36.31% of those enrolled; sampling error 4.18%, confidence level 95.5%, *Z* = 1.96, *p* = *q* = 0.5).

To develop competence in ethical and CSR issues in their students, instructors used a series of practical examples (case studies and readings) that were distributed throughout the semester via social networks. These materials incorporate analysis of some ethical and CSR aspects of specific ethical concerns in companies. To learn about the ethical dimensions of the subject, students first received a brief explanation during class time by the instructor about the objectives of a case or reading. They then were given time to document and work on the assignment before going on to solve it using social media tools. The instructor proposed cases of ethical issues related to firms that appeared on Twitter. These news resources show the company as an agent of social change in the sense that it employs a series of resources, interacts with society and cannot ignore the demands of the different stakeholders involved or affected by business activity, including the larger community. They also illustrate specific policies that the company could follow on social and environmental matters and suggest the possible effects of those policies on the company’s financial outcomes (mainly, the income statement and the ethical distribution of added value). Likewise, the role played by each stakeholder in the business activity was discussed on Facebook groups and forums, and students’ work was evaluated by other groups of students (García-Morales et al., 2020). The instructor concluded the discussion by explaining the reasoning behind each policy’s *raison d’être.* Finally, the final exam included ethical and CSR issues on which students had to comment.

### Measurements

A survey was carried out at two different points in time (the beginning and end of the semester) using a Likert rating scale (ranging from 1 to 5; value 1: strongly disagree, value 5: strongly agree; the questionnaire is in Annex).

Previous research in this field was taken into account in preparing the survey, which was divided into three parts to investigate students’ perceptions of a company’s CSR actions (*CSR perceptions*), the importance of the different CSR strategies that it carried out (*CSR commitment*) and the reasons why it chose to act in a socially responsible way (*CSR reasons*; see Annex). Specifically, the following variables were measured:

*CSR perceptions*: The scale proposed by Abdul and Ibrahim (2002) was used to measure ethical perceptions of society. The questionnaire included six items.

*CSR commitment*: The variables that measured the company’s social commitment were obtained from the models proposed by Zahra and LaTour (1987), Kraft and Singhapakdi (1991), and Nga and Shamuganathan (2010), with a total of 21 items.

*CSR reasons*: We based our reasons on the articles by Kha and Atkinson (1987), Pivo (2008), Brønn and Vidaver-Cohen (2009), and Pedersen and Neergaard (2009), which analyze these topics from the point of view of ethics and socially responsible action. The questionnaire included nine items.

To evaluate the fit of the measurement model, all constructs needed to have high internal consistency, as determined by their compound reliability (CR) and shared variance (SV) scores (Del Barrio and Luque, 2012; see Table 1).

**TABLE 1:** Measurement-model results.

Variables	α	C.R.	AVE
CSR perceptions	0.878	0.85	0.60
CSR commitment	0.892	0.81	0.50
CSR reasons	0.853	0.82	0.43

The constructs have satisfactory levels of confidence, as indicated by the composite reliability ranges (0.81–0.85) and ranges of SV coefficients (0.43–0.6). Likewise, for each factor, the composite reliability exceeded 0.70 and the average variance extracted (AVE) of 0.5 indicated good construct reliability (Hair et al., 2010). In addition, internal consistency and reliability were at a satisfactory level, as is reflected in the Cronbach’s alpha scores, and each factor presented a value above 0.8, reflecting good internal consistency. This led us to accept as valid the constructs used to define the variables of the model that we wanted to contrast.

### Research Methodology

Descriptive statistics were paired with a *t*-test to test the hypotheses. Previously, Levene’s test was used to check the equality of the variances. The *t*-test for independent samples is used to test a hypothesis of the equality of two means and is the most powerful test available when the normality of the sample is satisfied (Lehmann and Romano, 2005). Thus, in this article, we used the parametric test (*t*-test) to test the differences between pairs in order to determine whether the difference in means between the groups was statistically significant.

## Results

With regard to the first section of table in the Annex, which refers to students’ perceptions of ethics and CSR (*CSR perception*), the students thought that society’s highest ethical demands on companies are that they should meet society’s expectations and that ethical practices have an effect on a company’s image (items 4 and 6 had the highest averages, Annex). This seems to indicate that students have a perception that society demands that companies manage the interests of different stakeholders and that not doing so has a negative effect on their image. The perception that citizens do not feel defenseless against the actions of companies (the average for item 1 is the lowest in the block: 3.23 at the beginning and 3.36 at the end of the semester, Annex) validates this interpretation.

According to the data in this first section, except for questions 2 and 5, all other questions are significant. The values obtained at the end of the semester, however, are for the most part not greater than those that were obtained at the beginning (see Annex averages), which leads us to reject hypothesis 1. At the end of the semester, students perceived the power of the company to be greater than they had at the beginning, indicating their perception that society feels vulnerable to the actions of companies (the average at the end of the semester is 3.36 for item 1) since they believe that companies respond less to the legal requirements and demands of society and place less importance on the repercussions that their ethical actions may have on their image (higher averages at the beginning of the semester: 3.70 for item 3, 4.36 for item 4 and 4.34 for item 6, Annex). This indicates that students have preconceived ideas of ethics and CSR. It seems, therefore, that these initial thoughts are difficult to change or reinforce (Cohen et al., 2001), despite the use of Web 2.0 and social media tools in the teaching–learning process.

In the second section, with regard to *CSR commitment*, the aspects that scored highest were those related to the product and the employees (see Annex averages for items 22 to 27), followed by economic benefits and environmental factors (see Annex averages for items 8–10 and 12). For students, the company’s CSR commitment is related to the nature of the company’s main activity.

It seems that students do not place much importance on the company’s role as a social agent or in promoting ethical solutions (low mean scores for items 24–26, 30, and 31). It is here, however, that, together with the employees’ aspects, the main significant differences are found (see Annex, items 13–15, 19, and 20), which would lead us to partially accept hypothesis 2. The results show that after training in ethics and CSR, there is variation in students’ perceptions of the company’s commitment to social issues (Holland and Albrecht, 2013; Rodriguez-Gomez et al., 2020).

Finally, with respect to the third section (*CSR reasons*), students consider the companies’ motives to act ethically to be for the sake of the company’s reputation, image and leadership, as well as to respond to society’s demands and to achieve success and profit (where averages were 4.18/4.00 for item 28, 4.10/4.02 for item 31, 3.98/3.87 for item 34 and 3.95/3.83 for item 35). The perception of what they think society demands from companies, which we analyzed in the first block, coincides with the reasons they think justify the ethical thinking of companies.

It is evident that, according to the students, the reasons for companies’ ethical behavior are utilitarian, since the ethical behavior of the companies is linked to their reputation, image and success; it is intended to help obtain benefits rather than to act altruistically. In behaving ethically, their aim is simply to avoid the negative consequences of unethical behavior (Cacioppe et al., 2008). Nevertheless, the students understand that acting according to ethical and CSR criteria is a way of participating in society by satisfying the interests of stakeholders and that the success of the company depends on its ethical commitment (items 29, 34, and 36) (Holland and Albrecht, 2013), and this leads us to accept hypothesis 3 (see Annex).

## Discussion and Conclusion

This article aimed to analyze students’ perceptions of business ethics and CSR when using Web 2.0 and social media tools to develop ethical competence.

The results show that students perceive that companies try to respond to society’s ethical demands and that ethical practices have an effect on companies’ images (Brunton and Eweje, 2010). They also believe that the main reasons for a company’s carrying out CSR policies are for purposes of reputation, image, leadership, success, and profit (Hosmer, 2006).

As students are trained in CSR, however, they become more aware of the power of companies, perceiving society as having less ability to demand that a company meets society’s expectations and demands. This finding shows that students do not have a clear notion of the ethical requirements and legitimate interests of society that a company should respect and serve (Barnett et al., 1994).

When students delimit a company’s ethical commitments toward society, they highlight aspects related to the employees and the products, as well as the pursuit of economic benefit without philanthropic commitments. Therefore, students have a reduced vision of the ethical commitments of companies, and instructors should be required to introduce more cases that lead students to consider a broad model of ethics that is in line with the current demands and requirements of the market and society (Triadó et al., 2014). The training of the students, however, has led to changes, especially in relation to the commitments that the company must assume regarding social aspects (Shrivastava, 2010; García-Morales et al., 2020). This finding supports the applicability of Stakeholders Theory.

Therefore, in relation to the main contribution of our article—the effect that Web 2.0 tools and social media have on the perception of companies’ CSR commitments—we must highlight several conclusions. To get started, these tools do not seem sufficient to change students’ previous conceptions of business ethics and CSR. The adoption of collaborative and interactive methodologies affects their perceptions of the social commitments that companies must assume (Aral et al., 2013), but it does not provide the broad vision of ethics that CSR entails. This finding indicates the first implication of this study, which is the need to use other teaching methodologies in addition to those examined here. It appears that on social issues, through their use of social media, students come to understand the important role that companies play in society. This finding is in line with previous research (López-Pérez et al., 2011; García-Morales et al., 2020). We understand that continuing to work along these lines and through these methodologies can lead us to see that the company really is an active participant that can facilitate social change in all its aspects. Another research implication is that this methodology is appropriate to develop the students’ social vision of companies. The results indicate that this type of creative, flexible and collaborative methodology, where communication and interaction are encouraged and which develops emotions, curiosity, and a critical sense, is suitable for the development of ethical competence and can address current problems in the field of business (Berthon et al., 2012).

These conclusions lead us to ask ourselves questions that can inform future research. In view of the results, the research has several implications related to two areas that can strengthen the development of ethical competence. First, analysis of the answers provides the instructor with evidence to guide the discussion of the cases toward critical aspects that must be addressed. Second, it is necessary to do more work with the material provided prior to the discussions and to encourage student participation in the forums to give continuity to the student experience so that they can continue working on these ethical considerations throughout their undergraduate studies (Serrano and Pons, 2011). The fact that the experience was developed in first-year students who are only taught introductory material may partially explain the results. This constitutes the main limitation of the research.

In this sense, and as a future line of research, we would like to contrast the results of our survey with an examination of students in their final year to analyze the effect of these tools on students’ learning of various areas of CSR across their undergraduate experience. At the same time, we would like to incorporate other tools, such as the development of an app, the creation of a wiki or complementary concept maps.

## Data Availability Statement

The raw data supporting the conclusions of this article will be made available by the authors, without undue reservation.

## Ethics Statement

Ethical review and approval was not required for the study on human participants in accordance with the local legislation and institutional requirements. Written informed consent from the patients/participants or patients/participants legal guardian/next of kin was not required to participate in this study in accordance with the national legislation and the institutional requirements.

## Author Contributions

All authors listed have made a substantial, direct and intellectual contribution to the work, and approved it for publication.

## Conflict of Interest

The authors declare that the research was conducted in the absence of any commercial or financial relationships that could be construed as a potential conflict of interest. The handling Editor declared a shared affiliation with one of the authors with the authors at the time of review.

## Annex

**Table:**

	*t*-test		Mean	Mean
	*t*	Significance (two-tailed)	Beginning	End
**FIRST SECTION (H1): CSR PERCEPTION**				
1. The average citizen feels helpless against the actions of the company	−2.2	0.028**	3.23	3.36
2. The company is critical of the ethical performance of most companies	1.47	0.146	3.43	3.33
3. Legal requirements influence the ethical behavior of firms	1.81	0.069*	3.7	3.58
4. Organizations must act in a manner consistent with the expectations of society on ethical issues	2.88	0.004**	4.36	4.17
5. Whenever companies generate an acceptable return, they have a social responsibility that goes beyond the interests of shareholders/owners	−0.84	0.399	3.67	3.73
6. Ethical practices have an effect on the image that society has of the organization	1.71	0.087*	4.34	4.15
**SECOND SECTION (H2): CSR COMMITMENT**				
**Environment**				
7. Create a company that respects the environment	−0.782	0.434	3.34	3.41
8. Conserve resources and recycle materials	−0.415	0.678	4.03	4.06
9. Programs implement for pollution control and waste reduction	0.078	0.938	4.07	4.06
10. Programs implement for energy saving and reduction of water consumption	0.301	0.764	4.26	4.24
**Economic**				
11. Promotion of a balance among economic, social and environmental interests	−0.098	0.922	3.19	3.2
12. Economic benefits	−0.956	0.34	4.52	4.57
**Social**				
13. To be able to identify social needs clearly	−2.53	0.012**	2.89	3.23
14. To be committed to a social vision	−2.84	0.005**	2.58	3.06
15. To be highly motivated to defend social needs	−5.38	0.000**	2.52	3.04
16. To improve long-term social needs	−0.276	0.783	3.31	3.34
17. To promote a balance between social mission and social values	1.675	0.094	3.25	3.1
18. To improve long-term quality of life	0.637	0.524	3.6	3.5
**Sustainability**				
19. To be an agent of social change	2.63	0.009**	2.91	3.16
20. To promote ethical solutions	24.23	0.000**	2.25	2.73
21. To perform philanthropy	−0.709	0.479	3.4	3.44
**Workers/employees**				
22. Training and employee training	2.66	0.008**	4.45	4.57
23. Improvement of quality and safety at work	1.285	0.199	4.57	4.51
24. Employee satisfaction and integration	1.993	0.047**	4.52	4.6
**Customer/product**				
25. Improvement of the quality and safety of the product/service provided	−0.522	0.602	4.6	4.63
26. Provision of adequate information about the product/service provided	−0.27	0.787	4.35	4.37
27. Improvement after sales service and customer complaints	−0.447	0.655	4.28	4.3
**THIRD SECTION (H3): CSR REASONS**				
28. To improve the reputation and image of the company	0.616	0.538	4.18	4
29. To participate in society	1.788	0.074*	3.77	3.81
30. To identify with ethical problems	−0.958	0.338	3.71	3.77
31. To obtain a competitive advantage	1.098	0.273	4.1	4.02
32. To comply strictly with current legislation	−0.371	0.71	3.32	3.36
33. Through innovation themes	−0.976	0.329	3.37	3.44
34. To meet the demands of society	1.826	0.068*	3.87	3.98
35. To act ethically and affect profit	−0.076	0.039	3.83	3.95
36. Because the success of the company depends on the existence of an ethical commitment	2.55	0.011**	3.75	3.92

*Significance levels: *p < 0.10, **p < 0.05, **p < 0.01.*
